# METTL3 promotes IL‐1β–induced degeneration of endplate chondrocytes by driving m6A‐dependent maturation of miR‐126‐5p

**DOI:** 10.1111/jcmm.16012

**Published:** 2020-10-23

**Authors:** Liang Xiao, Quanlai Zhao, Bo Hu, Jing Wang, Chen Liu, Hongguang Xu

**Affiliations:** ^1^ Reseach center of Spine Surgery Key Laboratory of Non‐coding RNA Transformation Research of Anhui Higher Education Institution (Wannan Medical College) Department of Spine Surgery Yijishan Hospital The First Affliated Hospital of Wannan Medical College Wuhu China

**Keywords:** degeneration, endplate chondrocytes, IL‐1β, METTL3, miRNA

## Abstract

METTL3 is an important regulatory molecule in the process of RNA biosynthesis. It mainly regulates mRNA translation, alternative splicing and microRNA maturation by mediating m6A‐dependent methylation. Interleukin 1β (IL‐1β) is an important inducer of cartilage degeneration that can induce an inflammatory cascade reaction in chondrocytes and inhibit the normal biological function of cells. However, it is unclear whether IL‐1β is related to METTL3 expression or plays a regulatory role in endplate cartilage degeneration. In this study, we found that the expression level of METTL3 and methylation level of m6A in human endplate cartilage with different degrees of degeneration were significantly different, indicating that the methylation modification of m6A mediated by METTL3 was closely related to the degeneration of human endplate cartilage. Next, through a series of functional experiments, we found that miR‐126‐5p can play a significant role in IL‐1β–induced degeneration of endplate chondrocytes. Moreover, we found that miR‐126‐5p can inhibit the PI3K/Akt signalling pathway by targeting PIK3R2 gene, leading to the disorder of cell vitality and functional metabolism. To further determine whether METTL3 could regulate miR‐126‐5p maturation, we first confirmed that METTL3 can bind the key protein underlying pri‐miRNA processing, DGCR8. Additionally, when METTL3 expression was inhibited, the miR‐126‐5p maturation process was blocked. Therefore, we hypothesized that METTL3 can promote cleavage of pri‐miR‐126‐5p and form mature miR‐126‐5p by combining with DGCR8.

## 1 INTRODUCTION

At present, spinal degenerative disease is one of the most common causes of a declining labour force and quality of life globally. Thus, it brings a very heavy economic burden not only to families, but also to society.^1^, ^2^, ^3^ Disc degeneration is the pathological basis of the occurrence and development of spinal degenerative diseases. Therefore, it is particularly important to understand the pathophysiological process of disc degeneration and actively take effective intervention measures to prevent and treat disc degeneration and related diseases.^4^ Endplate cartilage, a thin layer of transparent cartilage between the intervertebral disc and adjacent vertebral body, plays important roles in nutrient exchange in the nucleus pulposus, stress buffering of the intervertebral disc and maintenance of the normal shape of the adjacent vertebral body.^5^, ^6^, ^7^ Previous studies have shown that endplate cartilage degeneration is one of the causes for disc degeneration, and maintenance of the normal function of endplate cartilage plays a decisive role in the prevention and treatment of disc degeneration.^8^

Inflammatory responses are one of the most important inducing factors for the degeneration of endplate chondrocytes. First, they affect the quality of endplate cartilage matrix by regulating matrix synthesis efficiency and the activity of proteases. In addition, they regulate the growth, differentiation and apoptosis of chondrocytes through the cytokine network.^9^, ^10^ Interleukin‐1β (IL‐1β) plays an important regulatory role in inflammatory factors. Increased expression of IL‐1β can inhibit chondrocyte proliferation, destroy their balance of synthesis and catabolism, and induce the production of matrix metalloproteinases. Subsequently, this leads to degradation of the cartilage matrix, inhibition of collagen alpha‐1 (II) chain (COL2A1) and aggrecan (ACAN) synthesized by chondrocytes, and promotion of the formation of collagen alpha‐1 (I) chain (COL1A1), which has the characteristics of fibroblasts and thus promote chondrocyte denaturation and apoptosis.^11^ Therefore, understanding the molecular mechanism of IL‐1β–induced degeneration of endplate chondrocytes and searching for target molecules with regulatory functions may be the key to delay or reverse the degeneration of endplate chondrocytes.

In recent years, RNA post‐transcriptional modification and regulation has become a hot topic in the research of various diseases.^12^ m6A methylation, the most abundant endogenous RNA modification, is widely found in eukaryotic mRNAs.^13^ A large number of studies have shown that methylation of m6A plays a key role in the pathogenesis and development of diseases by regulating expression of target genes at the post‐transcriptional level (including translation and alternative splicing of mRNA and the maturation of microRNA).^14^ At present, few studies have investigated the relationship between methylation of m6A and cartilage degeneration.^15^ Moreover, it is unclear whether m6A methylation plays an important role in IL‐1β–induced degeneration of endplate chondrocytes. In this study, we found that the m6A methyltransferase METTL3 can interact with DGCR8 to regulate pri‐miR‐126‐5p and play a contributory role in IL‐1β–induced endplate cartilage degeneration. In addition, high expression of METTL3 was positively correlated with the degeneration degree of endplate cartilage, suggesting that METTL3 might be an important target for the prevention and treatment of endplate cartilage degeneration.

## 2 MATERIALS AND METHODS

### 2.1 Tissue sample acquisition and detection of m6A methylation level

One hunred and twenty six samples of endplate cartilage were collected from 69 patients who underwent cervical fusion operation (28 for single segmental fusion, 25 for double segmental fusion and 16 for three segmental fusion) in our unit. Patient details are shown in Table 1. Evaluation and grading were made according to Thompson's gross morphological grading method.^16^ Histologic grading: Grade I, endplate cartilage is transparent and uniform in thickness; Grade II, thickness of endplate cartilage is irregular; Grade III, local defect of endplate cartilage can be seen, indicating uneven; Grade IV, fibrocartilage appears in endplate cartilage, and subchondral bone is irregular and locally sclerotic; and Grade V, endplate cartilage is widely sclerotic. The study was approved by the ethics committee of Yijishan Hospital of Wannan Medical College (Wuhu, China). The Ethics number was 201514. All patients were aware of this study and signed the informed consent.

**TABLE 1:** Patient demographic data

Parameter	Value
Sex ratio (M: F)	50/19
Age, mean (range), years	52.86 ± 11.47(22‐75)
Operation level	
C3/4	19
C4/5	64
C5/6	43

Considering the individual differences, only one segment of cartilage sample was randomly selected from each patient for histological detection, the remaining 57 samples were for cell culture experiment. The methylation level of total mRNA in 69 endplate cartilage tissues was measured by colorimetry immediately after acquisition. Poly(A) and mRNA were purified with a GenElute mRNA Mini Prep Kit (Sigma‐Aldrich). An m6A RNA Methylation Quantification Kit (Epigenetek) was used for m6A RNA methylation quantification.

### 2.2 Safranin O staining

Sixty nine endplate cartilage specimens were fixed, embedded and sectioned in the sagittal plane with a thickness of 5 μm. After gradient dewaxing, paraffin sections were incubated with Safranin O staining solution (Gibco, Thermo Fisher Scientific), and histological changes were observed under a light microscope after clearing.

### 2.3 Primary cell culture, identification and IL‐1β treatment

Fifty seven cartilage tissue samples were transferred to a super‐clean platform quickly, and the cartilage was cut into fragments of about 1 mm^3^. Cartilage was collected into a centrifuge tube and digested with a series of 0.25% trypsin and 0.2% collagenase type II (Gibco, Thermo Fisher Scientific) at 37°C for 30 minutes and 3 hours, respectively. After the digestion was stopped and the supernatant was removed by centrifugation, Dulbecco's Modified Eagle's Medium with F‐12 Supplement (Gibco, Thermo Fisher Scientific) containing 15% foetal bovine serum (Gibco, Thermo Fisher Scientific) was added for cell counting. The cell concentration was adjusted to 2 × 10^5^ cells/mL, transferred into a 25‐mL culture bottle and cultured in a 37°C 5% CO_2_ incubator. Undigested cartilage was digested again with 0.2% type II collagenase, and cells were extracted and cultured in the same way. Five days later, monolayer cells were passaged when they reached approximately 80% confluency.

Cell growth was observed and photographed with an inverted phase‐contrast microscope. Cells were washed three times with phosphate‐buffered saline (PBS), fixed with 4% paraformaldehyde for 30 minutes and washed again with PBS. Next, 500 μL toluidine blue was added for 30 minutes, 500 μL alcian blue was added for 60 minutes, and then, the cells were washed again with PBS after removing the dye with a pipette. After natural drying, cells were sealed with neutral gum and then observed and photographed under an inverted microscope.

Passage‐two cells in good growth condition were seeded into 96‐well plates at a density of 2000 cells per well. IL‐1β (Gibco, Thermo Fisher Scientific, Waltham, MA, USA) was diluted to 5 ng/mL and 10 ng/mL in PBS, and then added into the 96‐well plates (each concentration was added in three wells); blank control wells were also set up. Cells were cultured in the incubator for 2 days for subsequent detection.

### 2.4 Detection of cell proliferation and apoptosis

To assay cell proliferation, treated chondrocytes were seeded into 96‐well plates at a density of 5 × 10^3^ cells per well. Freshly prepared Cell Counting Kit‐8 solution (Dojindo Molecular Technologies, Tokyo, Japan) 10 μL was added to the plates containing cells for incubation at 37°C for 2 hours. After seed, the plates were vortexed for 2 minutes and the absorbance of each well was measured at 450 nm using a spectral scanning multimode microplate reader (Bio‐Rad).

For analysis of apoptosis, 195 μL of annexin V‐FITC binding solution (Beyotime Institute of Biotechnology, China) was added to treated endplate chondrocytes. Next, the cells were gently suspended and mixed with 5 μL of annexin V‐FITC (Beyotime Institute of Biotechnology, China), followed by 10 μL of PI staining solution (Beyotime Institute of Biotechnology, China) for seed in the dark at 25°C. Finally, the cells were put into an ice bath, and staining was examined by flow cytometry (BD Biosciences). Apoptosis of chondrocytes in endplate cartilage was analysed by FlowJo software (TreeStar).

### 2.5 Gene transfection

Target‐specific siRNAs were cloned into a lentivirus vector and transfected into endplate chondrocytes. Transfected cells were screened by puromycin (4 μg/mL) and then amplified to form stable sublines, which were used as a negative control (siCtrl). RT‐qPCR and Western blot were used to detect the knockout efficiency. The METTL3 siRNA sequence used in this study was GCTGCACTTCAGACGAATTAT. The wild‐type (WT) METTL3‐coding sequence (CDS) expression plasmid was generated by cloning the full‐length open reading frame of Sus scrofa METTL3 gene (XM_003128580.5). The DGCR8 siRNA sequence used in this study was GGAUGUAAAGAUUAGCGUGdTdT. miR‐126‐5p mimics and miR‐126‐5p inhibitor were both purchased from Guangzhou Ribobio Biotechnology and transfected using Lipofectamine 2000 (Invitrogen).

### 2.6 Reverse transcription quantitative polymerase chain reaction (RT‐QPCR)

According to the manufacturer's instructions, total RNA in endplate chondrocytes was extracted with TRIzol (Invitrogen). miRNA and mRNA expression levels were detected with a Bulge‐Loop^TM^ miRNA qRT‐PCR Starter Kit (Guangzhou Ribobio) and SYBR1 Premier Ex Taq^TM^ Kit, respectively. U6 snRNA and GAPDH were used as internal controls, respectively. Results were analysed with the relative quantitative 2^‐△△CT^ method. All experiments were repeated three times. Primer sequences are shown in Table 2.

**TABLE 2:** Primer sequences

Gene	Primer	Primer sequence (5′→3′)
METTL3	F	ATCCCCAAGGCTTCAACCAG
METTL3	R	GCGAGTGCCAGGAGATAGTC
DGCR8	F	CAAGCAGGAGACATCG GACAAG
DGCR8	R	CACAATGGACATCTTGGGC TTC
miR‐126‐5p	F	CGACGTCGTACCGTGAGT
miR‐126‐5p	R	CAGTGCAGGGTCCGAGGTAT
pri‐miR‐126‐5p	F	CGGGGTCCTGTCTGCATCCA
pri‐miR‐126‐5p	R	GTCTCAGCGGCGTTTTCGATG
U6	F	CGAGCACAGAATCGCTTCA
U6	R	CTCGCTTCGGCAGCACATAT
ACAN	F	CATTCACCAGTGAGGACCTCGT
ACAN	R	TCACACTGCTCATAGCCTGCTTC
COL2A1	F	TGAGGGCGCGGTAGAGACCC
COL2A1	R	TGCACACAGCTGCCAGCCTC
GAPDH	F	GCTGAGAACGGGAAGCTTGT
GAPDH	R	GACTCCACGACGTACTCAGC

### 2.7 Western blot

Proteins in endplate chondrocytes were lysed and collected, and the protein concentration was determined by the bicinchoninic acid quantitative method. Denatured protein was separated by sodium dodecyl sulphate‐polyacrylamide gel electrophoresis at 20 g per pore and then transferred onto a nitrocellulose membrane. The membrane was blocked with 5% bovine serum albumin for 1 hour, and primary antibodies against METTL3 (1:1000; Abcam), IL‐1β (1:100, Abcam), cleaved caspase 3 (1:100, Abcam), cleaved caspase 9 (1:100, Abcam), cleaved poly(ADP‐ribose) polymerase (PARP; 1:500, Abcam), ACAN (1:100, Abcam), COL2A1 (1:5000, Abcam), GAPDH (1:5000, Abcam), PIK3R2 (1:5000, Abcam), pAKT (1:5000, Abcam), AKT (1:500, Abcam) or DGCR8 (1:1000, Abcam) were added. The membrane was then incubated at 4°C overnight. The following day, a secondary antibody (1:5000; Cell Signaling Technology, Danvers, MA) was added and the membrane was incubated again for 1 hour at room temperature on a shaker. After washing the membrane three times with Tris‐buffered saline containing Tween 20, images were acquired with a gel imaging system.

### 2.8 Immunohistochemical staining

Paraffin sections of cartilage tissue were prepared, embedded, trimmed and sliced with a vibratome into 5‐μm sections. After drying, dewaxing and gradient alcohol hydration, antigen repair was carried out. Anti‐IL‐1β (1:100, Abcam) was added and incubated overnight in a 37°C incubator. After washing with Tris‐buffered saline, the secondary antibody was added, and DAB staining and haematoxylin re‐staining were performed.

### 2.9 Cellular immunofluorescence

Endplate chondrocytes were fixed with 2 mL 4% paraformaldehyde, incubated with 2 mL 0.3% Triton X‐100 to permeabilize membranes, and then blocked with 100 μL 2% sheep serum blocking solution. ACAN (1:200, Abcam), COL2A1 (1:250, Abcam) and METTL3 (1:1000, Abcam) antibodies were added and incubated overnight at 4°C. The following day, 100 μL fluorescent secondary antibody was added and incubated for 30 minutes at room temperature in dark, and 100 μL DAPI was added and incubated for 5 minutes at room temperature in dark. After washing with PBS, content of the target protein was observed with a laser‐scanning confocal microscope (Leica).

### 2.10 Luciferase reporter assay

The 3'‐untranslated region sequence (3'‐UTR) of the PIK3R2 gene was amplified by PCR using 293T genomic DNA as a template and cloned into the pmiR‐RB‐REPORT^TM^ double luciferase reporter vector as the wild‐type vector; the mutant vector was similarly constructed. Fluorescence of the vector resulted from the renilla luciferase gene (hRluc), while the firefly luciferase gene (hLuc) was used as an internal reference gene. 10 μL OPTI‐MEM medium was used to dilute miR‐126‐5p mimics or Non‐target Control(NC), 15 μL OPTI‐MEM medium was used to dilute the wild‐type vector or the mutant vector, and 25 μL OPTI‐MEM medium was diluted with 0.25 μL LipofectamineTM 2000 reagent. After 5 minutes, the above three reagents were mixed in order, each of which was 50ul. 293T cells were seeded into 96‐well plates with 1.5 × 104 cells per well. The above four mixtures were added in turn, and the total volume of each well was 100 μL. Luciferase substrate 35 μL/well and Stop reagent 30 μL/well were added in turn to measure the fluorescence value. All the above reagents were purchased from Guangzhou Ribobio.

### 2.11 CO‐immunoprecipitation (CO‐IP)

According to the instructions of a Pierce^TM^ Co‐IP Kit (Thermo Fisher Scientific), endplate chondrocytes were treated with lysate buffer. The obtained lysate products were divided into two groups. One group was labelled ‘input’ and used for Western blot to evaluate the protein content of METTL3 and DGCR8 at the end of the experiment. Lysates from the other group were mixed with an anti‐METTL3 antibody (1:50, Abcam). The antigen‐antibody complexes were collected after incubation, and expression levels of METTL3 and DGCR8 were detected by Western blot. Normal rabbit IgG was used as a negative control antibody.

### 2.12 Statistical analysis

SPSS 18.0 (IBM) was used for statistical analysis. All measurement data were normally distributed. Comparisons between the two groups of measurements were analysed by independent‐sample *t* test. Differences between three or more groups were analysed by one‐way analysis of variance. Tukey Honest Significant Difference test was used to assess the homogeneity of variance, while Dunnett's T3 test was used to assess the heterogeneity of variance. Pearson analysis was used to test the correlation between two groups. Differences were considered statistically significant if *P* < .05.

## 3 RESULTS

### 3.1 Degree of endplate chondrocyte degeneration correlated with levels of m6A methylation and METTL3 expression

**FIGURE 1:**
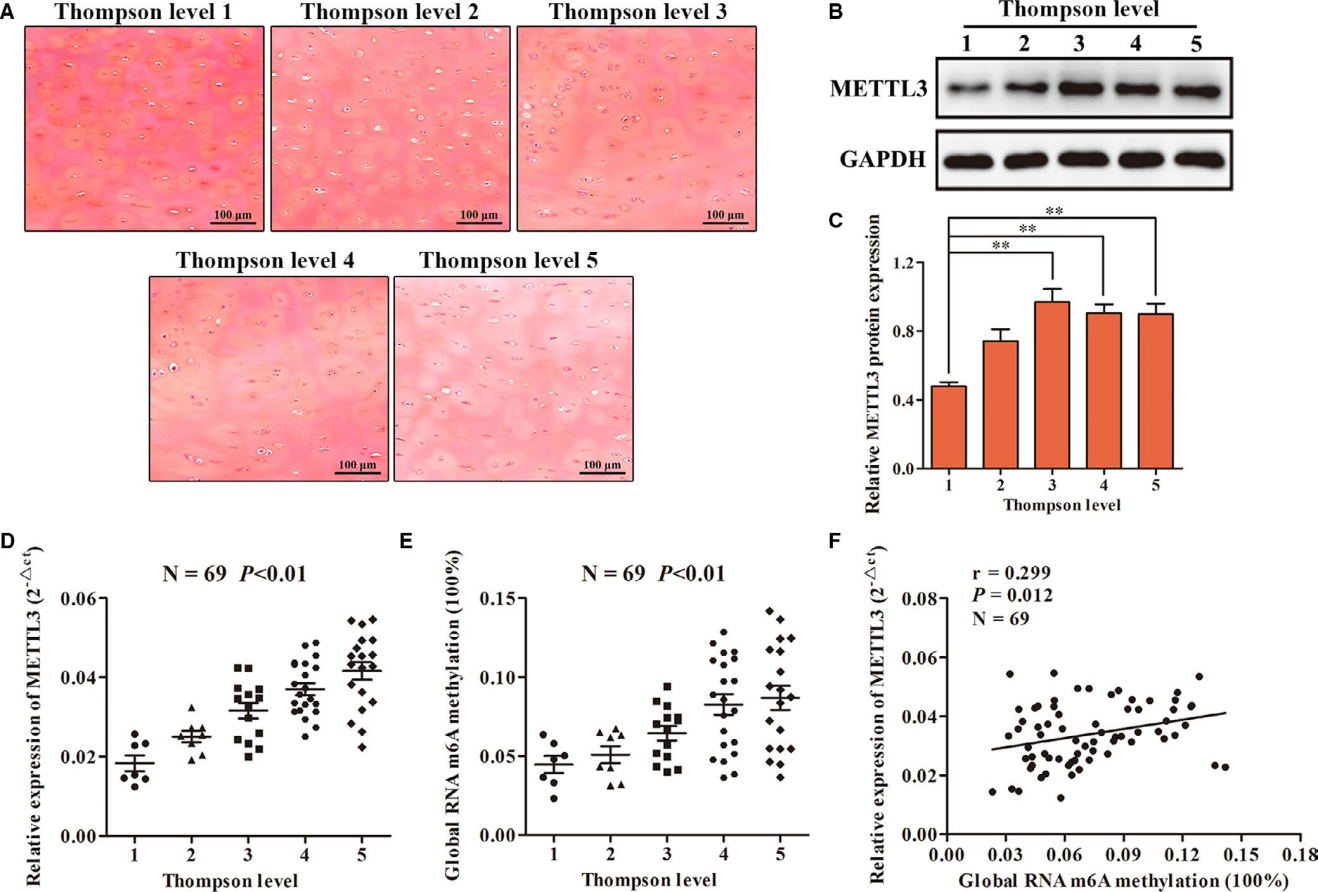
The difference of m6A methylation and METTL3 expression in 69 human endplate cartilage. A, Saffron O staining confirmed that the above level I‐V degenerative endplate cartilage had different pathological changes. B, C, Western blot was used to detect the difference of METTL3 protein expression among the five groups. D, RT‐qPCR was used to detect the difference of METTL3 expression in the five groups. E, The difference of global methylation level of m6A in the five groups was detected by colorimetry. F, Pearson test was used to analyse the correlation between the expression level of METTL3 and m6A methylation level. (**P < .01)

According to Thompson's gross morphological classification, 69 human endplate cartilage tissues were divided into Grade I (7 cases), Grade II (8 cases), Grade III (14 cases), Grade IV (21 cases) and Grade V (19 cases). It was observed by safranin O staining that chondrocytes were evenly distributed in the cartilage lacuna, and the cartilage matrix was uniformly stained red in the cartilage tissue of Grade I endplate. The distribution of chondrocytes in Grade II and Grade III endplate cartilage was a little disordered: some chondrocytes in the cartilage lacunae were hypertrophied, and red staining of the cartilage matrix was lighter compared with Grade I. The distribution of chondrocytes in Grade IV and Grade V endplate cartilage was disordered, and apoptosis and hypertrophy were observed. The red staining of cartilage matrix was obviously reduced compared with the former three groups (Figure 1A). RT‐qPCR, Western blot and colorimetry showed that with the gradual deterioration of endplate cartilage, expression of the m6A RNA methyltransferase METTL3 was increased, the overall methylated level of m6A was increased, and the two showed a low degree of positive correlation (Figure 1B–F). These results indicate that METTL3‐mediated methylation of m6A occurred in human endplate cartilage.

### 3.2 IL‐1β can induce degeneration of endplate chondrocytes

**FIGURE 2:**
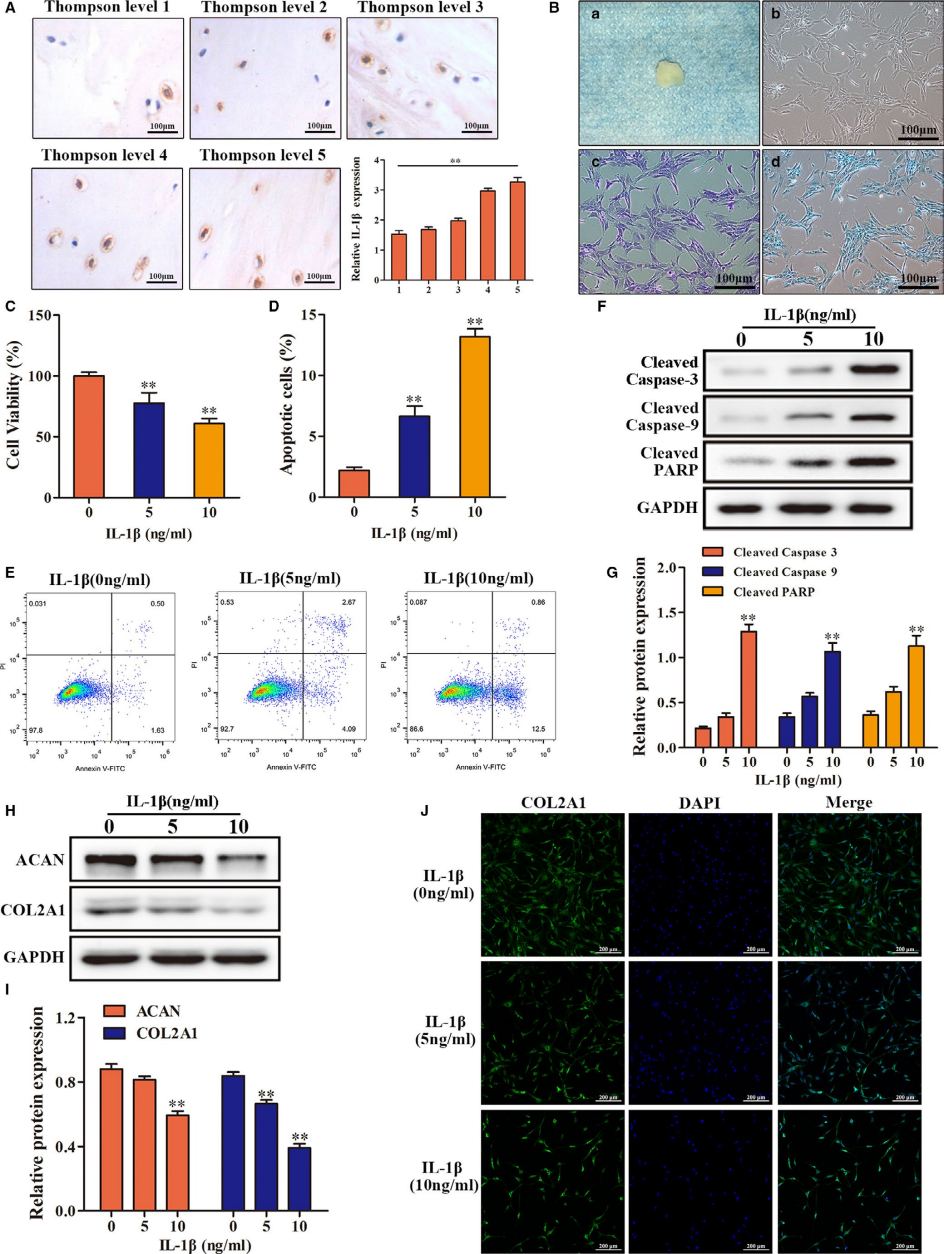
Effects of IL‐1β on human endplate chondrocytes. A, Immunohistochemistry was used to detect the IL‐1β expression in endplate cartilage with different degrees of degeneration. B, Culture and identification of human primary endplate chondrocytes. a, gross appearance of human endplate cartilage. b, microscopic appearance of human primary endplate chondrocytes. c, toluidine blue stain. d, alcian blue staining. C, The effect of different concentrations of IL‐1β on the proliferation of endplate chondrocytes. D, E, Annexin V‐FITC/PI was used to detect the effect of IL‐1β on the apoptosis of endplate chondrocytes. F, G, The effect of different concentrations of IL‐1β on the expression of apoptosis‐related proteins in endplate chondrocytes was detected by Western blot. H, I, J, Western blot and immunofluorescence were used to detect the effect of different concentrations of IL‐1β on the expression of phenotype protein in endplate chondrocytes. (**P < .01)

Immunohistochemistry was used to detect IL‐1β in human endplate cartilage with different degrees of degeneration. Using image J software to analyse the percentage of IL‐1β protein staining part in the whole image area, we found that the higher the degree of degeneration, the higher the expression level of IL‐1β in cartilage tissue (Figure 2A), suggesting that IL‐1β may play an important role in the degeneration of endplate cartilage. Next, we digested the human endplate cartilage and extracted primary cells for culture (Figure 2B). In our previous study, we observed the effects of different concentrations of IL‐β (0 ng/mL, 5 ng/mL, 10 ng/mL, 15 ng/mL and 20 ng/mL) on chondrocytes. The results showed that IL‐β more than 10 ng/mL was prone to a large number of cell death. We speculate that this may be due to the low viability of cells derived from human degenerative endplate. Therefore, in this study, we only observed the effects of IL‐β on chondrocytes at 0 ng/mL, 5 ng/mL and 10 ng/mL. We found that a high concentration of IL‐1β could significantly inhibit the proliferation of chondrocytes and induce apoptosis (Figure 2C‐G). In addition, a high concentration of IL‐1β inhibited the synthesis and metabolism of chondrocytes in the endplate, resulting in significantly down‐regulated contents of differentiation phenotypic proteins secreted by chondrocytes (Figure 2H‐J). Collectively, these results indicated that IL‐1β can induce the degeneration of chondrocytes in endplate.

### 3.3 Inhibition of METTL3 significantly reduced IL‐1β–induced degeneration of endplate chondrocytes

**FIGURE 3:**
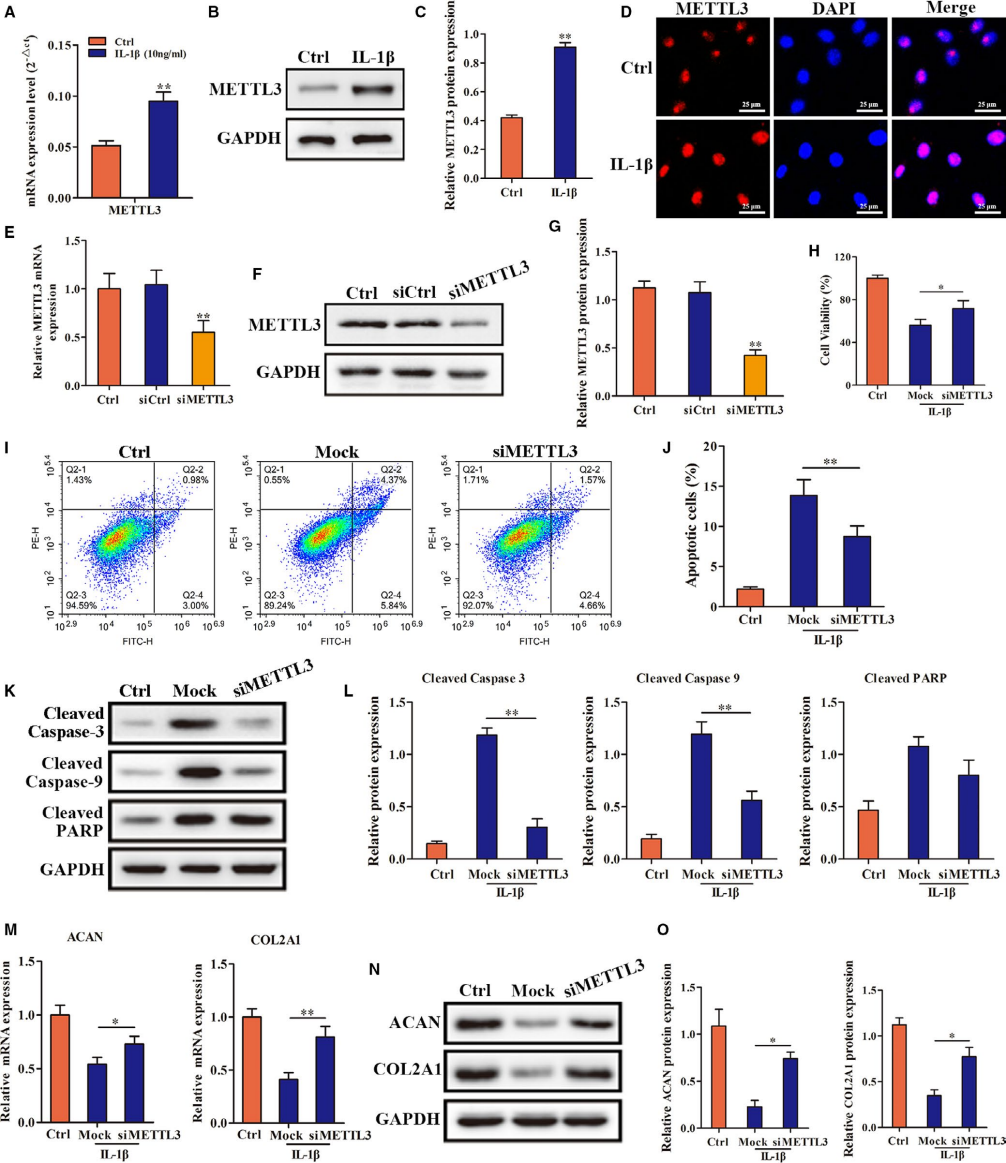
The regulatory role of METTL3 in IL‐1β induced degeneration of endplate chondrocytes. A, B, C and D, RT‐qPCR, Western blot and immunofluorescence were used to detect the expression of METTL3 in endplate chondrocytes under treatment of IL‐1β. E, F and G, The knockdown efficiency of METTL3 was detected by RT‐qPCR and Western blot. H, The effect of inhibition of METTL3 on the proliferation of endplate chondrocytes under treatment of IL‐1β. I, G, K and L, Annexin V‐FITC/PI and Western blot were used to detect the effect of inhibition of METTL3 on the apoptosis and related proteins expression in endplate chondrocytes under treatment of IL‐1β. M, N and O, RT‐qPCR and Western blot were used to detect the effect of inhibition of METTL3 on the expression of phenotype genes and proteins in endplate chondrocytes under treatment of IL‐1β. (*P < .05, **P < .01)

To investigate whether METTL3‐mediated m6A methylation participated in and regulated the pathological process of IL‐1β–induced degeneration of endplate chondrocytes, we examined METTL3 expression in endplate chondrocytes. The results showed that METTL3 expression in endplate chondrocytes was significantly up‐regulated in response to IL‐1β (Figure 3A‐D). Subsequently, we constructed a METTL3‐knockdown cell line (Figure 3E‐G) and found that when METTL3 expression was inhibited, IL‐1β treatment significantly ameliorated the inhibited proliferation and increased apoptosis of endplate chondrocytes (Figure 3H‐L). In addition, the impaired ability of endplate chondrocytes to secrete matrix protein was also significantly restored (Figure 3M‐O). These results indicated that inhibition of METTL3 significantly alleviated IL‐1β–induced degeneration of endplate chondrocytes.

### 3.4 MIR‐126 inhibited the PI3K/AKT pathway by down‐regulating PIK3R2 expression in response to IL‐1β

**FIGURE 4:**
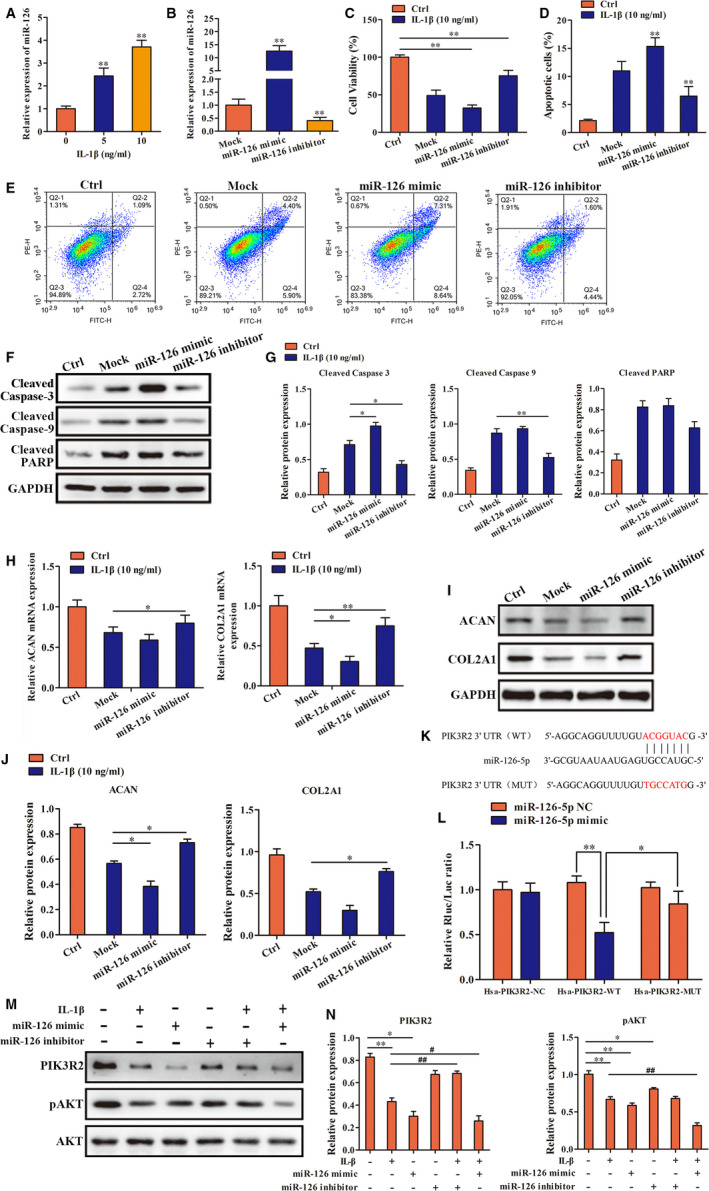
The mechanism of miR‐126 in IL‐1β induced degeneration of endplate chondrocytes. A, RT‐qPCR was used to detect the expression of miR‐126 in endplate chondrocytes under treatment of IL‐1β. B, The transfection efficiency of miR‐126 was detected by RT‐qPCR. C: The effect of regulation of miR‐126 on the proliferation of endplate chondrocytes under treatment of IL‐1β. D, E, F and G, Annexin V‐FITC/PI and Western blot were used to detect the effect of regulation of miR‐126 on the apoptosis in endplate chondrocytes under treatment of IL‐1β. H, I and J, RT‐qPCR and Western blot were used to detect the effect of regulation of miR‐126 on the expression of phenotype genes and proteins in endplate chondrocytes under treatment of IL‐1β. K, Diagrams of miR‐126‐5p and PIK3R2 binding sequences and the mutated sequence. L: Luciferase reporter assay. M and N: Changes in the expression of PIK3R2 and pAKT under the effects of IL‐1β and regulation of miR‐126 were detected by Western blot. (*P < .05, **P < .01, #*P* < .05, ##*P* < .01)

Previous studies have shown that METTL3 regulates miR‐126 maturation, so we examined the role of miR‐126‐5p in IL‐1β–induced degeneration of endplate chondrocytes and its molecular mechanism. First, we found that IL‐1β induced up‐regulation of miR‐126‐5p expression (Figure 4A). Next, we examined the effect of IL‐1β on endplate chondrocyte degeneration by overexpression and inhibition of miR‐126‐5p expression levels. The results showed that overexpression of miR‐126‐5p significantly aggravated the degeneration of endplate chondrocytes, while inhibition of miR‐126‐5p alleviated degeneration (Figure 4B‐J). Using target gene prediction, we identified PIK3R2 (also known as p85 β), the regulatory subunit of PI3K, to be a downstream target gene of miR‐126‐5p (Figure 4K). We constructed PIK3R2 wild‐type and mutant vectors, and the results of a luciferase reporter assay demonstrated that miR‐126‐5p could bind to the 3'UTR of the PIK3R2 gene (Figure 4L). Subsequent validation experiments showed that miR‐126‐5p was up‐regulated in endplate chondrocytes in response to IL‐1β inhibition of the PI3K/Akt pathway by targeted inhibition of PIK3R2 expression (Figure 4M, N).

### 3.5 METTL3 regulated MIR‐126‐5p maturity

**FIGURE 5:**
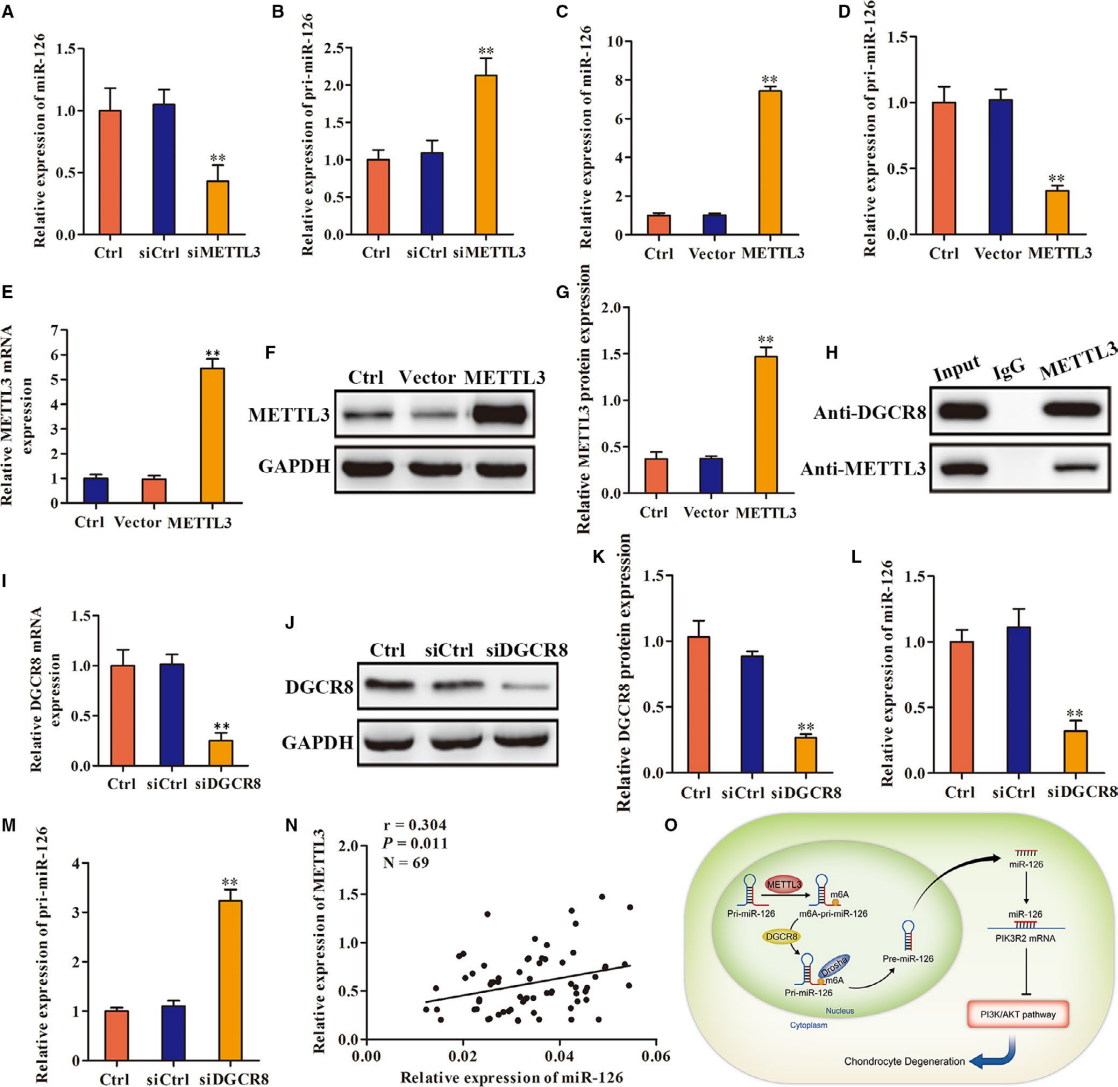
The effect of methylated modification mediated by METTL3 on the maturation of mir‐126‐5p. A and B, RT‐qPCR was used to detect change of miR‐126 and pri‐miR‐126 expression after METTL3 inhibition. C and D, RT‐qPCR was used to detect change of miR‐126 and pri‐miR‐126 expression after METTL3 overexpression. E, F and G, The overexpression efficiency of METTL3 was detected by RT‐qPCR and Western blot. H, Co‐IP was used to detect the combination between METTL3 and DGCR8. I, J and K, The knockdown efficiency of DGCR8 was detected by RT‐qPCR and Western blot. L and M, RT‐qPCR was used to detect change of miR‐126 and pri‐miR‐126 expression after DGCR8 inhibition. N, Pearson test was used to analyse the correlation between the expression level of METTL3 and miR‐126. O, Diagram of the mechanism. (**P < .01)

Finally, we examined whether METTL3 can regulate the formation and maturation of miR‐126‐5p. We observed that after METTL3 inhibition, miR‐126‐5p expression was significantly decreased and pri‐miR‐126‐5p expression was significantly increased (Figure 5A, B). On contrary, after METTL3 overexpression, miR‐126‐5p expression was significantly increased and pri‐miR‐126‐5p expression was significantly decreased (Figure 5C‐G). Using immunoprecipitation, we found that METTL3 can bind to DGCR8 protein (Figure 5H). In addition, DGCR8 inhibition was consistent with the result of METTL3 inhibition, miR‐126‐5p expression was significantly decreased and pri‐miR‐126‐5p expression was significantly increased (Figure 5I‐M). Besides, we examined human endplate cartilage and found that the expression level of METTL3 was positively correlated with miR‐126‐5p (Figure 5N). These results indicated that METTL3 was involved in pri‐miR‐126‐5p processing and promoted miR‐126‐5p maturity.

## 4 DISCUSSION

Gene expression is dynamic and reversible. It depends on transcriptional‐level regulation and, more importantly, post‐transcriptional modification of RNA.^17^ m6A RNA methylation is the most common and abundant eukaryotic mRNA post‐transcriptional modification.^18^ The methylation modification of m6A mainly involves three processes: RNA methylation mediated by METTL3/14, WTAP, RBM15/15B, VIRMA and ZC3H13, RNA demethylation mediated by FTO, ALKBH5, and ALKBH3, and downstream RNA translation and degradation involving YTHDF1/2/3 and ELAVL1.Using transcriptome sequencing, Chen et al^19^ found that METTL3 was highly expressed in human hepatoma cells, whereby it was highly related to the poor prognosis of hepatoma patients; SOCS2 gene was identified as a causal target of METTL3‐mediated m6A modification. Niu et al^20^ found that the m6A demethylase fat mass and obesity‐associated protein (FTO) promoted proliferation, colony formation and metastasis of human breast cancer cells; BNIP3 is a downstream target of FTO‐mediated m6A modification. At present, few studies have investigated the relationship between methylation of m6A and cartilage degeneration. In this study, we found that there was a significant difference in the level of m6A methylation in human endplate cartilage with different degrees of degeneration; that is, as the severity of degeneration increased, so did levels of m6A methylation and expression of the m6A methyltransferase METTL3. As METTL3 is a key component of the methyltransferase complex, we considered METTL3‐mediated m6A methylation to be closely related to the development of endplate cartilage degeneration, which may be an important target for the prevention and treatment of endplate cartilage degeneration.

IL‐1β is one of the most important mediators between the body and external stimuli. Previous studies have shown that increased IL‐1β production is closely related to a variety of diseases, including lupus arthritis, osteoarthritis and ankylosing arthritis.^21^, ^22^ IL‐1β has also been shown to be an important factor for intervertebral disc degeneration, whereby it stimulates a series of biochemical immune responses that cause dysfunction of the largest avascular tissue in the body, the intervertebral disc.^23^ In this study, we added IL‐1β to human cervical endplate chondrocytes cultured in vitro and found that it significantly inhibited cell viability, induced cell apoptosis and inhibited the normal secretory function of cells, resulting in cell degeneration consistent with previous research.^24^ Liu et al^25^ examined expression levels of METTL3 mRNA, m6A, and inflammatory cytokines in IL‐1β treated chondrocytes and found that both the level of METTL3 mRNA and percentage of m6A methylation were significantly increased. Their results indicated that METTL3 may play a functional role in osteoarthritis by regulating the nuclear factor κB signalling pathway, extracellular matrix synthesis and metabolism in chondrocytes. Therefore, to further determine whether stimulation with IL‐1β can affect METTL3 expression in endplate chondrocytes, we carried out functional studies and found that METTL3 expression in endplate chondrocytes was significantly increased after treatment with IL‐1β. In addition, we found that inhibition of METTL3 expression significantly alleviated IL‐1β–induced cell degeneration. These results demonstrate that METTL3 expression was closely related to stimulation with IL‐1β. By inhibiting METTL3 expression, the anti‐inflammatory ability of endplate chondrocytes can be enhanced, which may delay or even reverse the degeneration of endplate chondrocytes.

Post‐transcriptional gene expression is regulated by miRNAs. A large number of studies have shown that miRNAs play an important role in cartilage degeneration and differentiation.^26^, ^27^, ^28^ In addition, a previous study showed that IL‐1β induces chondrocyte inflammation and up‐regulates miR‐126 expression. Moreover, inhibition of miR‐126 alleviates the inflammatory response and apoptosis induced by IL‐1β.^29^ In this study, we explored the mechanism of miR‐126‐5p in IL‐1β–induced degeneration of endplate chondrocytes. We first found that with increasing concentrations of IL‐1β, the expression level of miR‐126‐5p in endplate chondrocytes increased. Subsequent overexpression and inhibition experiments showed that up‐regulation and down‐regulation of miR‐126‐5p aggravated and alleviated IL‐1β–induced degeneration of endplate chondrocytes, respectively. To further reveal the molecular mechanism of miR‐126‐5p, we employed bioinformatics and found that miR‐126‐5p has a binding site on the PIK3R2 gene. PIK3R2 is a member of PI3K p85 subunit family. As a regulatory subunit, PIK3R2 can significantly inhibit the activation of PI3K/Akt pathway. Previous studies have shown that PI3K/Akt signalling pathway can directly induce chondrocyte apoptosis and inhibit chondrocyte proliferation.^30^, ^31^ Furthermore, Zhang et al^32^ found that PI3K pathway can regulate extracellular matrix synthesis of rat endplate cartilage. A luciferase reporter assay showed an interaction between miR‐126‐5p and the 3'‐UTR of the PIK3R2 gene. As one of the target genes of miR‐126‐5p, PIK3R2 binds to miR‐126‐5p and causes downstream effects. To further determine whether miR‐126‐5p plays a role in the degeneration process of endplate chondrocytes through its target gene PIK3R2, we detected expression levels of PIK3R2 and its downstream regulatory molecule pAkt after overexpression and inhibition of miR‐126‐5p, respectively. The results showed that miR‐126‐5p regulated the expression of PIK3R2 and pAkt. In summary, these data indicated that IL‐1β promotes up‐regulation of miR‐126‐5p expression, which subsequently inhibits the PIK3R2‐mediated PI3K/Akt pathway.

Although the results described above reveal the mechanism of miR‐126‐5p in the process of IL‐1β–induced degeneration of endplate chondrocytes, the relationship between METTL3 and miR‐126‐5p in endplate chondrocytes in response to IL‐1β stimulation remains unclear. Recent studies have shown that metabolism and biological processes of miRNA are regulated by m6A methylation.^33^, ^34^ For example, researchers found that METTL3 can promote the maturity of miRNAs (such as miR‐126, miR‐221/222 and miR‐93) through m6A modification.^35^ In addition, recent studies have shown that methylated RNA is easier to cut and process into pre‐miRNA after binding with DGCR8.^36^, ^37^ This suggests that METTL3 may play a role in regulating miR‐126‐5p maturation under the action of IL‐1β. To test this hypothesis, we first examined whether METTL3 binds to the key protein of pri‐miRNA processing, DGCR8. Co‐IP results showed that METTL3 binds DGCR8. We further found that inhibition of METTL3 significantly reduced expression of miR‐126‐5p, while expression of pri‐miR‐126‐5p was significantly increased, indicating that the lack of METTL3 prevented cleavage of pri‐miR‐126‐5p.

## 5 CONCLUSION

In brief, this study revealed the interaction among METTL3, miR‐126‐5p and PIK3R2 in endplate chondrocytes under the action of IL‐1β (Figure 5O). Specifically, we found that IL‐1β up‐regulated METTL3 expression in endplate chondrocytes. Also, METTL3 promoted the up‐regulation of miR‐126‐5p expression by m6A methylation modification, and subsequently suppressed the protective effect of the PI3K/Akt pathway on endplate chondrocytes through its target gene PIK3R2, resulting in dysfunctional cell vitality and metabolism, and ultimately the degeneration of endplate chondrocytes.

## CONFLICT OF INTEREST

No conflict of interest exists in the submission of this manuscript, and manuscript is approved by all authors for publication. All the authors listed have approved the manuscript that is enclosed.

## AUTHOR CONTRIBUTIONS

**Liang Xiao:** Conceptualization (lead); Funding acquisition (lead); Writing‐original draft (lead); Writing‐review & editing (supporting). **Quanlai Zhao:** Data curation (lead); Investigation (equal); Software (lead). **Bo Hu:** Data curation (supporting); Software (supporting). **Jing Wang:** Data curation (equal); Software (supporting); Supervision (lead). **Chen Liu:** Data curation (lead); Resources (lead); Software (equal). **Hongguang Xu:** Conceptualization (lead); Funding acquisition (lead); Project administration (lead); Writing‐review & editing (lead).

## Ethical approval

All experimental procedures, specimen acquisition in this study was reviewed and approved by the ethics committee of Yijishan Hospital of Wannan Medical College (Wuhu, China). All surgical patients participating in the study were aware of the study and signed the informed consent forms.

## Data Availability

According to the requirements, data can be obtained from the corresponding authors to support the results of this study.
